# Survey of allele specific expression in bovine muscle

**DOI:** 10.1038/s41598-019-40781-6

**Published:** 2019-03-12

**Authors:** Gabriel M. Guillocheau, Abdelmajid El Hou, Cédric Meersseman, Diane Esquerré, Emmanuelle Rebours, Rabia Letaief, Morgane Simao, Nicolas Hypolite, Emmanuelle Bourneuf, Nicolas Bruneau, Anne Vaiman, Christy J. Vander Jagt, Amanda J. Chamberlain, Dominique Rocha

**Affiliations:** 1 GABI, INRA, AgroParisTech, Université Paris-Saclay, 78350 Jouy-en-Josas, France; 2 GMA, INRA, Université de Limoges, 87060 Limoges, France; 3 GenPhySE, Université de Toulouse, INRA, INPT, ENVT, 31326 Castanet Tolosan, France; 4 CEA, DRF/iRCM/SREIT/LREG, Jouy-en-Josas, France; 5 Agriculture Victoria Research, AgriBiociences Centre, Bundoora, Victoria, Australia

## Abstract

Allelic imbalance is a common phenomenon in mammals that plays an important role in gene regulation. An Allele Specific Expression (ASE) approach can be used to detect variants with a *cis*-regulatory effect on gene expression. In cattle, this type of study has only been done once in Holstein. In our study we performed a genome-wide analysis of ASE in 19 Limousine muscle samples. We identified 5,658 ASE SNPs (Single Nucleotide Polymorphisms showing allele specific expression) in 13% of genes with detectable expression in the *Longissimus thoraci* muscle. Interestingly we found allelic imbalance in *AOX1*, *PALLD* and *CAST* genes. We also found 2,107 ASE SNPs located within genomic regions associated with meat or carcass traits. In order to identify causative *cis*-regulatory variants explaining ASE we searched for SNPs altering binding sites of transcription factors or microRNAs. We identified one SNP in the 3’UTR region of *PRNP* that could be a causal regulatory variant modifying binding sites of several miRNAs. We showed that ASE is frequent within our muscle samples. Our data could be used to elucidate the molecular mechanisms underlying gene expression imbalance.

## Introduction

Gene regulation is a fundamental process in the development and maintenance of organisms. In mammalian genomes the variability of gene expression is a current phenomenon^1,2^. It is therefore important to study this variability in order to understand gene regulation. There are different approaches to such studies: expression quantitative trait loci (eQTLs) and Allele Specific Expression (ASE) analyses. The combination of both approaches is highly effective at locating *cis*- and *trans*- regulation of gene expression.

An expression quantitative trait locus (eQTL) is a DNA region with some nucleotide sequence differences (Single Nucleotide Polymorphisms, insertion, deletion) that affects the expression level of a gene in *cis* or *trans*. They can be identified by expression genome-wide association studies (eGWAS), an analysis method computing the likelihood of a polymorphism affecting gene expression. Unfortunately this type of analysis needs a large number of samples to minimize false-positives^3^. Many human eQTL mapping studies have been carried out^4–6^ including the recent Genotype-Tissue Expression (GTEx) project^7^. However in cattle there is a lack of studies. So far, there has been only one performed in dairy cattle, in Holstein-Friesians (HF), Jerseys (J) and HFxJ crossbreeds^8^.

Allele specific expression (allelic expression or allelic imbalance) analysis is a robust approach to quantify expression variation between the two haplotypes of a diploid individual distinguished by heterozygous sites^9^. This approach is complementary to identifying variants affecting gene expression with eQTL studies because we can use a smaller number of samples^10^. Genome-wide studies of ASE have been performed in different species (human^11^, mouse^12^ or fruit fly^13^) including livestock species (pig^14^, chicken^15^ or sheep^16^). In addition, some ASE genes were detected to impact economically important traits^10,17^.

In cattle, only two studies have been performed so far, both in Holstein. In the first study, they discovered 473 ASE SNPs across 5 bovine blastocysts (among 2,524 different heterozygous SNPs)^18^. In the second study, they detected 19,082 ASE SNPs (1,060 on average per tissue) across 18 different tissues from one lactating Holstein dairy cow^19^.

In our study, we performed a genome-wide investigation of ASE using 19 Limousine calf muscle samples. We distinguished between imprinting (parental mono-allelic expression) and allele specific expression (not mono-allelic expression) to focus on the later. We used whole-genome sequences (WGS) and RNA-Seq data from these 19 muscle samples in our analysis. To the best of our knowledge, it is the first ASE survey in a beef breed and with the largest number of different animals.

## Materials and Methods

### Animals and tissue samples

Nineteen Limousine bull calves were selected from a large study on the genetic determinism of beef and meat quality traits^20^. They were fattened in a single feedlot and fed *ad libitum* with wet corn silage. They were humanely slaughtered in an accredited commercial slaughterhouse when they reached 16 months. *Longissimus thoracis* (LT) muscle samples were dissected immediately after death and tissue samples were snap frozen in liquid nitrogen and then stored at −80 °C. The animals used in this study were beef animals raised for commercial reasons from a previous study^20^ and were slaughtered by certified slaughterhouses in accordance with French animal protection regulations (Code Rural, Articles R214-64 to R214-71; Legifrance, 2011).

### Whole-genome sequencing and sequence alignment

DNA was extracted from the 19 muscle samples using the Wizard Genomic DNA Purification kit (Promega). Each purified DNA sample was assessed by agarose gel electrophoresis. DNA concentration was measured with a Nanodrop ND-100 instrument (Thermo Fisher Scientific). Sequencing libraries were prepared using TruSeq SBS v3-HS Kit (Illumina) and the whole-genome sequenced using a 2 × 100  bp paired-end approach on an Illumina HiSeq2000. Sequence alignments were carried out using the Burrows-Wheeler Alignment tool (BWA-v0.6.1-r104)^21^ with the *aln* option with default parameters for mapping reads to the UMD3.1 bovine reference genome^22^. Potential PCR duplicates were removed using the MarkDuplicates tools from the Picard package version 1.4.0^23^. Only properly paired reads with a mapping quality of at least 30 (-*q* = 30) were retained. The resulting BAM files were then used for all subsequent analyses.

### RNA sequencing and sequence alignment

RNA extraction and sequencing was performed as previously described^24–26^. Briefly, after transfer to ice-cold RNeasy RLT lysis buffer (Qiagen), LT tissue samples were homogenized using a Precellys tissue homogeniser (Bertin Technologie). Total RNA was isolated using RNeasy Midi columns (Qiagen) and then treated with RNAse-free DNase I (Qiagen) for 15 min at room temperature according to the manufacturer’s protocols. The concentration of total RNA was measured with a Nanodrop ND-100 instrument (Thermo Scientific) and the quality was assessed with an RNA 6000 Nano Labchip kit using an Agilent 2100 Bioanalyzer (Agilent Technologies). All 19 samples had an RNA integrity number (RIN) value greater than eight.

The mRNA-Seq libraries were prepared using the TruSeq RNA Sample Preparation Kit (Illumina) according to the manufacturer’s instructions. Briefly, Poly-A containing mRNA molecules were purified from 4 μg total RNA of each sample using oligo (dT) magnetic beads and fragmented into 150–400 bp pieces using divalent cations at 94 °C for 8 min. The cleaved mRNA fragments were converted to double-stranded cDNA using SuperScript II reverse transcriptase (Life Technologies) and primed by random primers. The resulting cDNA was purified using Agencourt AMPure XP beads (Beckman Coulter). Then, cDNA was subjected to end-repair and phosphorylation and subsequent purification was performed using Agencourt AMPure XP beads. These repaired cDNA fragments were 3′-adenylated producing cDNA fragments with a single ‘A’ base overhung at their 3′-ends for subsequent adapter-ligation. Illumina adapters containing indexing tags were ligated to the ends of these 3′-adenylated cDNA fragments followed by two purification steps using Agencourt AMPure XP beads. Ten rounds of PCR amplification were performed to enrich the adapter-modified cDNA library using primers complementary to the ends of the adapters. The PCR products were purified using Agencourt AMPure XP beads and size-selected (200 +/− 25 bp) on a 2% agarose Invitrogen E-Gel (Thermo Scientific). Libraries were then checked on an Agilent Technologies 2100 Bioanalyzer using the Agilent High Sensitivity DNA Kit and quantified by quantitative PCR with the QPCR NGS Library Quantification kit (Agilent Technologies). After quantification, three different tagged cDNA libraries were pooled in equal ratios and a final qPCR check was performed post-pooling. Each library pool was used for 2 × 100 bp paired-end sequencing on one lane of the Illumina HiSeq2000 with a TruSeq SBS v3-HS Kit (Illumina). After sequencing, the samples were demultiplexed and the indexed adapter sequences were trimmed using the CASAVA v1.8.2 software (Illumina). The quality of the raw sequence reads was assessed using FastQC and Qualimap^27^.

The *Bos taurus* reference genome sequence was downloaded from Ensembl (release 91, Bos taurus-UMD3.1.dna.toplevel.fa). To align the reads to the assembled reference genome the STAR RNA-Seq (version 2.4.2a) aligner was used^28^. Default values were used for mapping except for the intron alignment (alignIntronMin: 20 and alignIntronMax: 500,000). Reads for each sample were mapped separately to the reference genome sequence. Only paired reads were retained for alignment. The number of paired-reads uniquely aligning to transcribed regions of each transcript was calculated for all genes of the annotated transcriptome. The transcript paired-read count was calculated as the number of unique paired-reads that aligned within the exons of each transcript, based on the coordinates of mapped reads.

### SNP identification and annotation

SNPs were called following the best practices from GATK (version 3.4–46) with HaplotypeCaller for DNA and RNA sequence data respectively^29,30^. First, reads were subjected to local realignment, coordinate sorting, base quality score recalibration and indel realignment. We then performed SNP and indel discovery and genotyping. In the GATK analysis, we used a minimum confidence score threshold of Q30 with default parameters. We also used multi-sample variant calling in order to distinguish between a homozygous reference genotype and a missing genotype among the analysed samples. SNPs were annotated with VEP^31^ using the transcript set from Ensembl 87.

### Detection of ASE SNPs

We used ASEReadCounter^9^ to calculate read counts per allele. We performed an N-masking (replacing for each identified variant the nucleotide of the bovine genome reference sequence by N) to remove mapping bias and we only kept overlapping heterozygous SNPs from DNA and RNA to remove discordant genotypes, possibly due to imprinting or RNA editing. We only kept candidates with minimum 10 reads for at least one allele. To determine if the imbalance was significant, we used a binomial test against an allelic ratio of 0.5 with a *p*-value of 5% (Python).

### Correlation analysis

The SNP being tested for ASE might not be the variant regulating the expression of the gene. So in order to determine the SNPs within the regulatory regions or potentially the regulatory variant itself, we detected SNPs in linkage disequilibrium with our ASE SNPs using PLINK 1.9^32^ (intra-chromosomal analysis and *r*^2^ > = 0.75). We used HTSeq-count^33^ to determine the number of reads for each transcript per individual and normalised this using DESeq2^34^. We computed the Spearman’s rank correlation coefficient between the genotypes of ASE SNPs or SNPs in LD and expression level of the corresponding transcript. We performed a correction for multiple testing, for the same transcript, using the Bonferroni correction.

### ASE SNP validation

First-strand cDNA was synthesized from 500 ng of DNase I-treated total RNA using the SuperScript III First-Strand Synthesis System kit (Thermo Fisher Scientific) and oligo-dT primers with random hexamers, according to the manufacturer’s instructions in a total volume of 20 μl. The resulting cDNA was diluted 1:10.

PCR and Pyrosequencing primers were designed using PyroMark Assay Design 2.0 (Qiagen) with sequences previously masked with RepeatMasker^35^. One of the forward or the reverse PCR primer had a 5′-biotin modification and was HPLC-purified. Primers were synthesized by IDT and are listed in Table S1. Polymerase chain reactions were performed in 50 μl using 1 μl of diluted cDNA or 100 ng of genomic DNA, 1 U GoTaq DNA polymerase (Promega), 1X PCR buffer, 1.5 mM MgCl_2_, 200 μM of each dNTP and 0.3 μM of each PCR primer. The following touchdown cycling protocol was used: 95 °C for 2 min, followed by 13 cycles of 95 °C for 1 min, 1 min of annealing (the annealing temperature was progressively lowered from 68 to 56 °C in steps of 1 °C every cycle) and 72 °C for 1 min 30 s. These initial cycles were followed by 20 cycles of 95 °C for 1 min, 55 °C for 1 min and 72 °C for 1 min 30 s, and a final extension step at 72 °C for 10 min. To check the quality of the amplification 10 μl of PCR products were then analysed by gel electrophoresis with a 1% agarose gel.

Biotinylated PCR products (20 μl) were immobilized on streptavidin-coated Sepharose beads (GE Healthcare), purified, washed and denatured using a 0.2 M NaOH solution and rewashed all using the PyroMark Vacuum workstation (Qiagen) as recommended by the manufacturer. Purified single-stranded PCR product was annealed to the pyrosequencing primer (diluted to 0.3 μM) and then sequenced using the PyroMark Q24 system (Qiagen), following the manufacturer’s instructions. For validating candidate ASE SNPs, DNA and RNA (cDNA) from each sample were pyrosequenced simultaneously. The proportions of individual alleles for each SNP were obtained using the PyroMark Q24 software version 1.0.10 (Qiagen). Genomic DNA was examined to confirm the heterozygosity. The final ASE ratio for each SNP of each sample analysed was calculated using the following formula: ASE ratio = (allele 1%/allele 2%) RNA/(allele 1%/allele 2%) genomic DNA.

### Prediction of microRNA binding sites

Prediction of microRNA (miRNA) binding sites was done as follows: first, for SNPs within 3′UTR regions, flanking sequences (+/−100 bases) were retrieved using the whole-genome reference sequence (UMD3.1). Then we created two versions of this sequence, one with the reference allele and one with the alternate allele. Next we used miRanda^36^ for both sequences with all known bovine miRNAs using the default parameters. Bovine miRNA sequences were retrieved from the miRBase database (version 21). To finish, we selected miRNAs which could bind only one of these two sequences.

## Results and Discussion

### DNA and RNA sequencing data statistics

Sequencing of all 19 whole-genome sequences generated a total of 5.3 billion of raw paired-end reads corresponding to 537.51 Gb. Approximately, 92 to 400 million paired-end reads were obtained for each library. On average, 83% (56–92%) of the paired-end reads were properly aligned with BWA on the UMD3.1 bovine reference genome (Table S2).

Sequencing of all 19 RNA-Seq libraries generated a total of 1.4 billion raw paired-end reads. Approximately, 35 to 180 million paired-end reads were obtained for each library. On average, 89% (86–91%) of the reads were uniquely mapped (Table S3). In a previous study^26^, 17 of our 19 RNA samples were sequenced and mapping was performed using BWA (version 0.5.9-r16)^21^. 63–76% of the mapped reads were aligned. The increase of the mapping rate (on average 17.8% more reads) indicates that STAR performs best. This is largely because STAR is a splice aware aligner. The mapping performance is comparable to other studies done in cattle with STAR and the same reference genome (UMD3.1). For instance 90% of transcripts from Holstein-Friesian peripheral blood leukocytes were mapped^37^.

The count of transcripts was performed using HTSeq-count^33^ and was normalized with DESeq2^34^. In our samples, we found 18,206 transcripts (corresponding to 16,338 genes) with an expression in at least 3 individuals among the 19.

### Variant detection

We identified 11,943,766 and 269,390 single nucleotide variants (SNVs) from WGS and RNA-Seq data, respectively.

We identified on average 11,344,542 +/− 7.12% SNVs per individual from WGS and on average 53,732 +/− 31.85% SNVs per individual from RNA-Seq reads. On average, 26.2% and 34.2% of the detected SNVs were heterozygous in WGS and RNA-Seq, respectively. Among the SNVs identified from WGS (Table 1), we identified 8,099,157 (67.81%), 2,922,660 (24.47%), 413,619 (3.46%), 405,237 (3.39%) as intergenic, intronic, upstream gene, downstream gene variants, respectively. We identified 69,096 (0.58%) exonic variants (56.62% synonymous, 43.32% missense and 0.07% coding sequence variants). For the other types of variants, the percentage was less than 0.20%: 19,332 3′UTR (0.16%) and 3,544 5′UTR variants (0.03%).

**Table 1:** Summary of SNPs detected in RNA and DNA with their annotation frequencies.

Variant consequences	DNA		RNA	
	Number of SNPs	%	Number of SNPs	%
intergenic variant	8,099,157	67.81	54,410	20.20
intron variant	2,922,660	24.47	106,700	39.61
upstream gene variant	413,619	3.46	14,734	5.47
downstream gene variant	405,237	3.39	53,630	19.91
synonymous variant	39,119	0.33	14,315	5.31
missense variant	29,931	0.25	9,786	3.63
3 prime UTR variant	19,332	0.16	11,555	4.29
splice region variant	6,471	0.05	475	0.18
non coding exon variant	3,930	0.03	0	0.00
5 prime UTR variant	3,544	0.03	1,374	0.51
unindentified	269	0.00	132	0.05
splice donor variant	153	0.00	73	0.03
splice acceptor variant	148	0.00	44	0.02
initiator codon variant	62	0.00	0	0.00
coding sequence variant	46	0.00	59	0.02
mature miRNA variant	37	0.00	0	0.00
stop retained variant	32	0.00	15	0.01
non coding transcript variant	19	0.00	11	0.00
frameshift variant	0	0.00	1,221	0.45
protein altering variant	0	0.00	1	0.00
non coding transcript exon variant	0	0.00	855	0.32

Among variants found with RNA-Seq data, we identified 54,410 (20.20%), 106,700 (39.61%), 14,734 180 (5.47%), 53,630 (19.91%) as intergenic, intronic, upstream gene, downstream gene variants, respectively. We identified 24,160 (8.97%) exonic variants (59.25% synonymous, 40.5% missense and 0.24% coding sequence variants).

We found 67.8% of SNPs from WGS data as intergenic. This percentage is in agreement with the 70.4% of the intergenic part of the bovine genome. This proportion is also similar in others studies done in cattle. For instance 73% of intergenic, 26.2% of intronic, 4.26% of downstream gene and 4.14% of upstream gene variants were found in Hanwoo and Yanbian cattle^38^ or 65.6% of intergenic and 33.6% were identified of intronic variants in Qinchuan cattle^39^. Interestingly, we found 20.20% (54,410) of SNPs identified from our RNA-Seq data as intergenic. These SNPs could be located in transcripts of large intergenic non-coding RNAs. Indeed, we found 7,706 (14,16%) intergenic SNPs from our RNA-Seq data within lincRNAs previously identified from six of our samples by Billerey and collaborators^25^. We also found 39.61% of SNPs identified from our RNA-Seq data in intronic regions. These SNPs could be from premature transcripts (before splicing).

### RNA-Seq and DNA-Seq SNP comparison

We compared SNPs detected from WGS with SNPs from RNA-Seq data for each individual. On average, we detected 11,306,326 SNPs only from WGS (out of 11,943,766 detected SNPs), 15,516 SNPs only from RNA-Seq reads (out of 269,390 detected SNPs), and 38,217 of the SNPs from both (Table 2). We focused on overlapping SNPs identified from WGS and RNA-Seq data and checked the concordance between their genotype. This overlap is on average 90% (75.7% to 96.0%) concordant (69% for both homozygous and 31% for both heterozygous). For the 10% discordant SNPs, 84.3% are homozygous from DNA-Seq and heterozygous from RNA-Seq data. This could be explained by RNA editing. 15.7% are heterozygous from DNA-Seq and homozygous from RNA-Seq; this could be explained by gene imprinting (mono-allelic expression). Alternatively, discrepancies between DNA and RNA genotypes could be due to sequencing errors. To study the allelic imbalance, we only kept the heterozygous concordant SNPs.

**Table 2:** Distribution of detected SNPs from RNA-Seq and WGS data per individual.

Individual	DNA only	RNA only	Overlap	BH	Bh	Concordant	Hh	hH	Discordant
LIM01	11,420,182	19,039	44,861	27,410	11,354	86.4%	4,979	1,118	13.6%
LIM02	11,549,679	17,681	46,624	29,535	12,671	90.5%	3,974	444	9.5%
LIM03	11,753,420	15,867	49,721	31,024	16,413	95.4%	1,633	651	4.6%
LIM04	11,770,633	13,801	38,579	23,198	12,968	93.7%	1,149	1,264	6.3%
LIM05	11,668,108	11,596	36,346	22,687	11,637	94.4%	1,513	509	5.6%
LIM06	11,645,235	16,568	44,888	27,925	12,860	90.9%	3,295	808	9.1%
LIM07	11,287,139	6,218	15,075	9,088	3,439	83.1%	1,947	601	16.9%
LIM08	11,734,961	18,876	55,713	35,061	17,430	94.2%	2,306	916	5.8%
LIM09	11,563,319	13,215	33,473	21,119	9,012	90.0%	2,897	445	10.0%
LIM13	8,718,858	27,165	28,651	18,020	3,671	75.7%	6,707	253	24.3%
LIM14	11,665,886	12,410	34,686	22,388	9,932	93.2%	1,796	570	6.8%
LIM15	11,516,569	15,344	40,398	25,775	10,135	88.9%	3,931	557	11.1%
LIM16	11,766,765	12,041	35,918	22,612	11,854	96.0%	890	562	4.0%
LIM17	9,511,239	21,194	28,415	17,675	3,677	75.1%	6,863	200	24.9%
LIM18	11,755,926	8,686	24,893	15,029	8,585	94.9%	902	377	5.1%
LIM19	11,517,295	15,901	40,528	25,083	11,315	89.8%	3,573	557	10.2%
LIM20	11,330,071	12,058	19,755	12,190	4,423	84.1%	2,753	389	15.9%
LIM21	11,110,581	14,100	30,031	19,059	6,466	85.0%	4,147	359	15.0%
LIM22	11,534,319	23,041	77,560	45,999	24,815	91.3%	5,907	839	8.7%
Average	11,306,326	15,516	38,217	23,730	10,666	89.1%	3,219	601	10.9%

BH: Both Homozygous, Bh: Both Heterozygous, Concordant: Rate of BH and Bh, Hh: Homozygous in DNA and Heterozygous in RNA, hH: Heterozygous in DNA and Homozygous in RNA, Discordant: Rate of Hh and hH.

### ASE SNP identification

**Figure 1:**
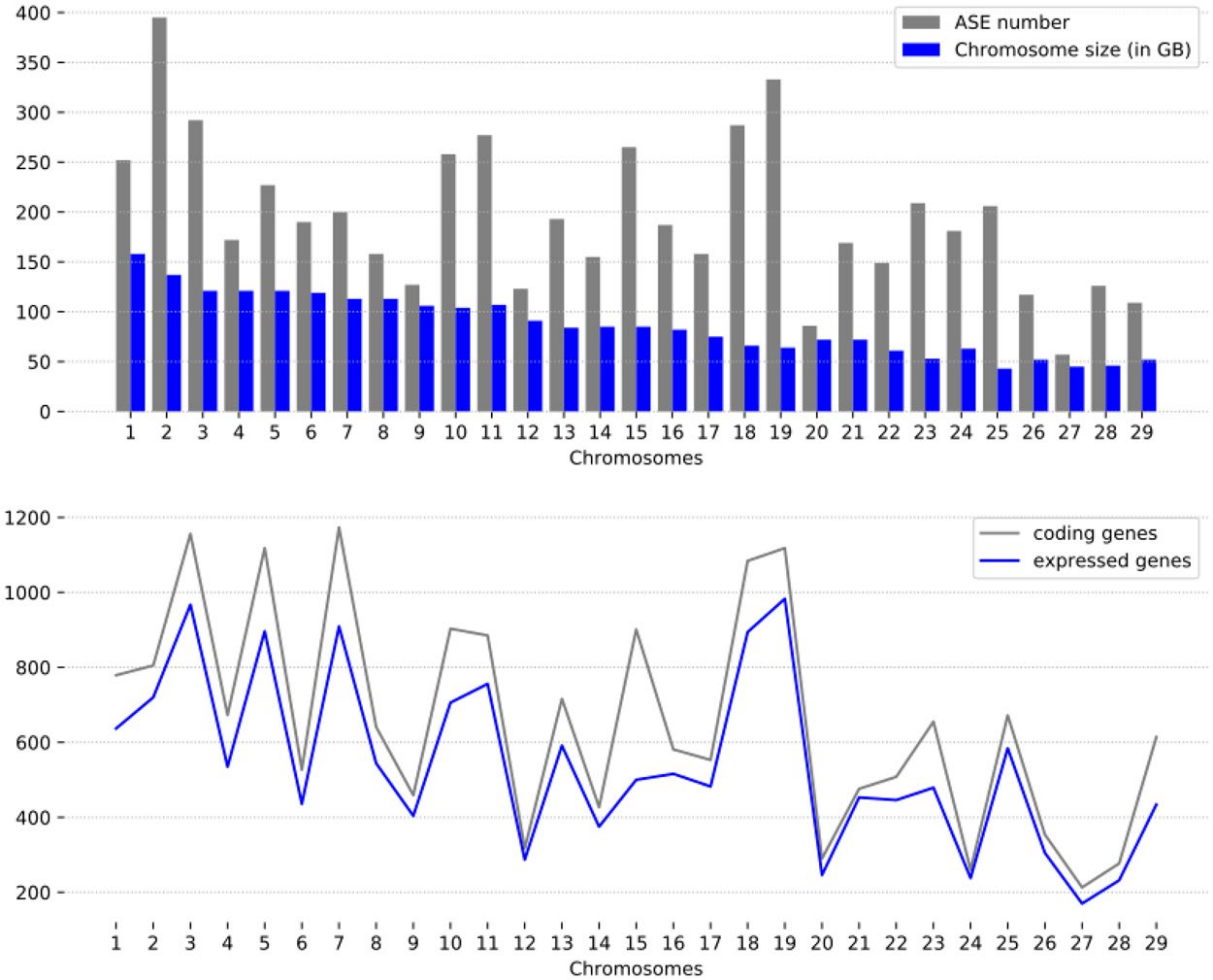
Chromosomal distribution with the number of ASE SNPs (grey bars), the size of the genomes (blue bars), the number of genes: total (blue line) and only expressed in muscle (grey line).

Using ASEReadCounter we calculated reads count per allele for all heterozygous concordant SNPs from alignment to the UMD3.1 reference genome sequence and the N-masked genome sequence. On average, the N-masking removed 27.1% of the candidate SNPs from ASE detection. We identified 6,908 ASE SNPs (Table S4) in 2,451 genes corresponding to 9.8% of all bovine genes (25,066), 15% of the genes with detectable expression in *Longissimus thoraci* muscle (16,338) and 20% of the genes with at least one heterozygous SNP (12,269). On average, we detected 574 ASE SNPs per individual (min: 184, max: 991) corresponding to 3.2% of the heterozygous SNPs from RNA-Seq data (Table S5). Last, we removed ASE SNPs within CNV regions previously identified within our Limousine animals^40^ and kept 5,658 ASE SNPs located in 2,119 genes. We then checked the distribution of the ASE SNPs across chromosomes. There is a weak correlation between the number of ASE SNPs per chromosome and the size of the chromosomes (*ρ* = 0:45, *p*-value = 0.015). However, the number of ASE SNPs per chromosome is strongly correlated with the number of coding genes (*ρ* = 0:84, *p*-value = 9.13 E-09) and with the number of expressed genes (*ρ* = 0:85, *p*-value = 4.81 E-09) (Fig. 1).

We compared our detected ASE SNPs with ASE SNPs previously identified by Chamberlain and collaborators in a Holstein muscle sample^19^. In their study, ASE detection was performed on one lactating dairy cow using TOPHAT2^41^ for the read alignment and a Chi-squared test. We found 118 ASE SNPs in common with the 2,006 ASE SNPs from Holstein muscle representing 5.9% of their detected ASE SNPs. We investigated why we do not detect the remaining ASE SNPs in our results. 684 of these SNPs (34.1%) were not polymorphic in our Limousine animals, 43 others SNPs (2.1%) are not showing heterozygosity among our 19 individuals and 38 SNPs (1.9%) are located on the chromosome X (excluded because we have only males). For the 1,123 remaining ASE SNPs (60.0%) identified in Holstein muscle, we found at least one heterozygous Limousine animal. This discrepancy might be due to differences in ASE detection methods or in breed gene regulation.

### Functional annotation of ASE SNPs and of their genes

4,193 of the detected ASE SNPs were located within cattle QTL regions reported in Animal QTLdb^42^ (Table S6). Interestingly, 1,213 of these ASE SNPs were inside QTL regions found in Limousine and 2,107 of these SNPs were in QTL regions linked to growth or meat traits.

In order to study the impact of genes affected by ASE on specific biological pathways, we performed a Gene Ontology (GO) enrichment. This analysis was carried out by first converting the cow gene list into a human gene list using Biomart^43^. This resulted in a list of 2,143 genes that was tested for enriched GO terms using the GOrilla tool^44^ with a background gene list of all expressed genes in *Longissimus thoraci* muscle (13,998).

**Figure 2:**
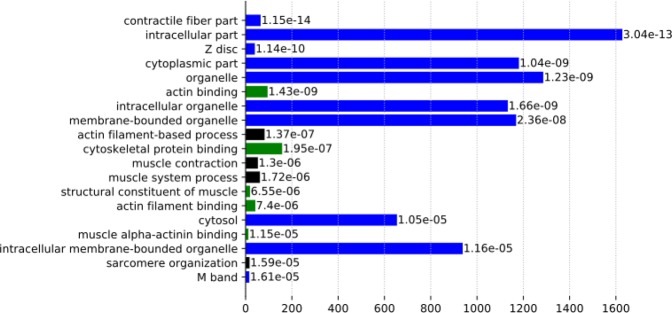
Enriched GO terms for genes affected by ASE. Functional enrichments for gene ontology (GO) terms associated with the 2,119 genes affected by ASE SNPs (5,658). Only the top ranked 20 terms are shown. The horizontal bar represents the number of ASE-genes involved, with the corresponding *q*-values. The GO terms categories included Biological Process (black), Cell Component (blue) and Molecular Function (green). The enrichment analysis was performed with the GOrilla tool.

In total, the genes showing ASE corresponded to 127 enriched functions (*q*-value < 0.05), with many of these related to striated muscle development (Table S7). The top 20 most-enriched terms are presented in Fig. 2. Thirteen functions were related to muscle functions or components: contractile fiber part (GO:0044449), Z disc (GO:0030018), actin binding (GO:0003779), actin filament-based process (GO:0030029), cytoskeletal protein binding (GO:0008092), muscle contraction (GO:0006936), muscle system process (GO:0003012), structural constituent of muscle (GO:0008307), actin filament binding (GO:0051015), muscle alpha-actinin binding (GO:0051371), sarcomere organization (GO:0045214) and M band (GO:0031430). The seven GO terms not directly related to muscle were linked to intracellular part and/or organelle and can be associated with contractile fibre part, mitochondrion or nucleus.

### ASE validation

We used Pyrosequencing in order to validate ASE SNPs. Several filters were applied to narrow down the number of ASE SNPs to test. Firstly, we kept ASE SNPs present in a QTL region associated with growth or meat quality traits reported in Animal QTLdb. Secondly, we removed SNPs absent from dbSNP. Then, we only kept ASE SNPs present in exonic, 5′UTR or 3′UTR regions. Finally, we selected two ASE SNPs located within *CAST* and we choose randomly four extra ASE SNPs.

**Figure 3:**
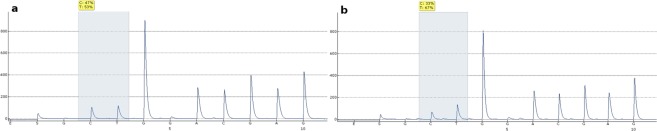
Pyrosequencing results of one ASE-SNP in *PALLD* gene. (**a**) In gDNA, 47% for allele C and 53% for allele T. (**b**) In cDNA, 33% for allele C and 67% for allele T.

We tested these 6 ASE SNPs by Pyrosequencing with replicates (Table 3). Technical replicates obtained from independent experiments show standard deviations ranging from 0–4% indicating that our Pyrosequencing procedure has negligible inter-PCR and Pyrosequencing variations. The allele frequencies determined for genomic DNA samples, which we analysed in duplicate showed an average variation of 2% +/− 1% (n = 4). For the cDNA samples, the average variation between replicates was 2% +/− 2% (n = 4).We could therefore detect allele frequency differences larger than 4%. Five ASE SNPs were validated by Pyrosequencing. For example, we observed for the validated ASE SNPs rs110694123 in *PALLD* gene 47% for allele G (complementary base of C) and 53% for allele A (complementary base of T) in gDNA and we observed 33% and 67% in cDNA (Fig. 3). We get an ASE ratio of 1.80 showing an allelic imbalance in favour of allele A (it means there is 1.80 more expression of transcripts with the A allele than with the G allele). This is consistent with the ASE ratio computed from the read counts for this SNP (1.52 with 39.67% for G and 60.33% for A).

**Table 3:** ASE SNPS tested by Pyrosequencing.

BTA	Position	SNP ID	REF	ALT	ASE count	Gene	Annotation	Validated
3	32,003,949	rs382378456	C	A	407/336	*ATP5F1*	3′UTR variant	Yes
7	5,520,428	rs208775256	G	C	26/12	*PGLS*	missense variant	No
7	98,579,574	rs41255587	G	A	146/208	*CAST*	3′UTR variant	Yes
7	98,580,401	rs209641420	A	C	303/221	*CAST*	3′UTR variant	Yes
8	572,167	rs110694123	G	A	48/73	*PALLD*	synonymous variant	Yes
8	944,049	rs109919583	C	T	47/121	*CBR4*	3′UTR variant	Yes

REF: reference allele, ALT: alternative allele, ASE count: number of reference allele reads/number of alternative allele reads.

### *Cis*-regulation of genes showing allele specific expression

Our detected ASE SNPs are probably not the causative variants, but rather markers in *cis* with the causative polymorphisms. It is known that the majority of causative SNPs are in regulatory regions instead of coding regions^45^. Therefore, we were looking for a link between ASE SNPs and the putative causative SNPs in *cis*. With this in mind, we used PLINK to identify all the SNPs in linkage disequilibrium (LD) (*r*^2^ > = 0.75) with our predicted ASE SNPs. We obtained 2,955 SNPs (including ASE SNPs) with genotypes for all the 19 individuals. For each transcript showing allele-specific expression, we calculated the Spearman correlation coefficient score between expression level of genes containing ASE SNPs and genotypes of SNPs in LD with ASE SNPs. We computed correlations between 2,794 SNP genotypes and 1,085 unique transcripts, averaging 2.74 SNP genotypes per transcript (min 1, max 37). We found 100 significant correlations with 45 transcripts (*ρ* > |0.6| and *q*-value < 0.05) including 42 negative correlations (Table S8). 25 of those correlations involved an ASE SNP.

**Figure 4:**
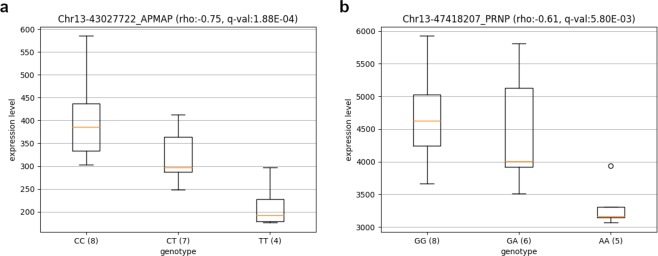
Boxplots of SNP showing genetic variations of *APMAP* (**a**) or *PRNP* (**b**) expressions. (N) number of animals per genotype.

For example, we found one SNP (C/T, rs41691181) in LD (*r*^2^ = 0.79, distance of 12.5 kb) with a SNP (C/T, rs208256739) in upstream and exonic (synonymous variant) regions of *APMAP* respectively. The second SNP shows ASE in one individual (LIM8) among the nineteen. The genotypes of the first SNP (8 C/C, 7 C/T, 4 T/T) is significantly correlated (*ρ* = −0.75 and *q*-value = 0.000188) to the *APMAP* level expression. Indeed, we found on average for the 19 animals 404, 323 and 214 transcripts (read counts) for C/C, C/T and T/T animals (Fig. 4a) showing an expression bias in favour of the C allele. We investigated how this SNP (rs41691181) in the upstream gene region could cause this allelic imbalance by testing if the SNP could alter Transcription Factor Binding site (TFBS) using TFBS-match^46^ with the SNP flanking sequences (+/−10 bases). None of the allele-specific sequences of these SNPs were located in predicted TFBS.

We extended the TFBS search for 5 other SNPs in 5 different genes (*5 S rRNA*, *LRRC66*, *ENSBTAG00000026637*, *GLOD4* and *PLK1*) with a significant correlation in the upstream region without detecting any TFBS.

In another example, we found one SNP (G/A, rs109763272) in LD (*r*^2^ = 0.86, distance of 274 bases) with a SNP (G/A, rs378125518). Both SNPs are in 3′UTR region of the *PRNP* gene and show ASE in four individuals among the nineteen. The genotypes of the first SNP (8 G/G, 6 G/A, 5 A/A) is significantly correlated (*ρ* = 0.61 and *q*-value = 0.0057966) to the *PRNP* expression level. On average, the *PRNP* expression level was 4,641 transcripts for G/G individuals, 4,455 for G/A individuals and 3,324 for A/A individuals (Fig. 4b) showing an expression bias in favour of allele G. Given that this correlated SNP is also an ASE SNP, we looked if allele counts estimated with ASEReadCounter is in agreement with the transcript expression level. Indeed, transcripts with the G allele are 1.54 times more expressed than transcripts with the A allele. We investigated how this SNP (rs109763272) in 3′UTR region could cause this allelic imbalance. It is known that polymorphisms in microRNA (miRNA) binding sites may affect miRNA/target gene interaction^47^. Therefore, we used miRanda to detect miRNA binding sites within this SNP flanking region. We predicted 9 miRNAs which could bind the reference allele (G) and 5 miRNAs which could bind the alternate allele (A) (Table S9). Interestingly, we noticed less expression with the alternate allele (Fig. 4b). This could suggest that some of the 5 detected miRNAs binding with the A allele could reduce the expression of *PRNP*.

We lack data on miRNA expression in our samples but several studies describing catalogs of miRNAs expressed in bovine muscle or skeletal muscle satellite cells have been published^48–58^. However, no study describes so far miRNAs expressed in Limousin animals. We found that all fourteen miRNAs impacted by the SNP rs109763272 are expressed in muscle^50–53^ including in *Longissimus dorsi*/*thoracis*^53^ (Table S10). We therefore cannot exclude any of the 5 miRNAs binding to the A allele or any of the 9 miRNAs binding to the G allele, as candidate *PRNP* regulators. Further work is needed to identify which if any of these candidate miRNAs reduce *PRNP* expression level.

We extended the miRNA binding sites prediction analysis to all SNPs with a significant correlation and located in a 3′UTR region (Table S9). We analysed 13 additional SNPs present in 6 other genes (1 SNP in *ANKRD*, 1 in *CCDC90B*, 2 in *FAM32A*, 2 in *TYK2*, 3 in *IMP3* and 4 in *TTC3*). We found no binding sites for 3 of these SNPs and for the remaining 10 SNPs we always found allele-specific binding sites for both alleles (Fig. S1) including 8 SNPs with a lower expression with the alternate allele. This could suggest that some of the detected miRNAs are binding with the alternate allele to reduce the gene expression. We found 2 SNPs with a lower expression of the reference allele. Similar to the alternate allele, the detected miRNAs binding with the reference allele could reduce gene expression. Survey of miRNAs expressed in bovine muscle allowed us to exclude only eleven miRNAs (Table S10). Further work is needed to identify which SNPs impact target sites of the remaining 386 miRNAs.

For most of the 45 genes for which we had a significant correlation between expression level and SNP (ASE SNP or SNP in LD with an ASE SNP) genotypes we couldn’t find SNPs altering TFBSs or the binding sites of miRNAs. It is therefore likely that epigenetic mechanisms might also play a role, rather than just *cis*-regulatory genetic variants (in TFBS or 3′UTR).

### ASE genes potentially involved in meat quality traits

The aldehyde oxydase 1 (*AOX1*) gene encodes a homodimeric protein, which produces hydrogen peroxide. In mouse, it is involved in myogenesis^59^. Therefore, it might play a role in muscle development in cattle. We detected eleven ASE SNPs in this gene with six also detected by Chamberlain and collaborators^19^. Among these 6 ASE SNPs, three had genotypes significantly correlated to the expression of this gene. In addition, we found 13 others SNPs in *AOX1* with significant correlation (Fig. S2).

The palladin (*PALLD*) gene encodes a cytoskeletal associated protein, which exists as multiple isoforms^60^. This actin associated protein plays a significant role in regulating cell adhesion and cell motility. It is also important for the early smooth muscle cell differentiation in mouse^61^. In cattle, palladin might play dual roles (positive and negative) in maintaining the proper skeletal myogenic differenciation^62^. We detected two ASE SNPs in this gene including one experimentally validated by Pyrosequencing. Interestingly, these SNPs are within a QTL region associated with average daily gain (ADG) trait in Hereford^63^.

The calpastatin (*CAST*) gene encodes an inhibitor of protease μ-calpain, which has a known effect on beef muscle tenderness variation^64^. Interestingly, a more recent study confirmed that *CAST* affected meet tenderness in *Longissimus* muscle in Limousine crossed-breed animals^65^. We detected seven ASE SNPs in this gene including two experimentally validated.

These 3 genes could be associated with meat quality and carcass traits. Interestingly, one of the ASE SNPs found in *AOX1* is a missense variant. This SNP (rs109201304) modifies a glycine residue into a cysteine amino acid and is located within a protein region conserved in mammals (Fig. S3). This residue (p.G1023C) lies within the substrate pocket subdomain IV of the large C-terminal domain which is important for substrate access and positioning but also in the dimerization of the two AOX1 monomeric subunits^66,67^. Several studies performed on AOX1 variants resulting from rat or human missense SNPs have shown that some of these SNPs increased or decreased the rate of superoxide radical production^68–71^. Further work is needed to investigate whether r109201304 can affect the catalytic activity of bovine AOX1.

We didn’t find any missense polymorphisms in *PALLD* and *CAST* but we identified several synonymous variants (2 in *PALLD* and 2 in *CAST*). They don’t alter the primary sequence of the corresponding proteins however it has been shown that codon usage can vary between genes and that this codon bias can affect RNA secondary structure, splicing and translation^72^. Further work is needed to investigate the phenotypic impact of these variants/genes.

### Biological relevance of allele specific expression in muscle

Overall we identified 5,658 ASE SNPs in 13% of genes (2,119) with detectable expression in *Longissimus thoracis* muscle. The high number of genes potentially impacted by allele-specific imbalance prompted us to investigate if some of these ASE SNPs could have a major impact on muscle biology.

First we looked if ASE SNPs could induce a gene loss-of-function. We didn’t find any ASE SNP that could create or remove stop codons and causing consequently protein truncations or changes in the open reading frame, respectively. However, we identified 14 ASE SNPs that according to the VEP annotation have or could perturb the splicing of the corresponding gene. Further work is needed to check this potential impact.

Second we investigated further the 421 missense ASE SNPs. According to the VEP annotation, only 37 of those missense ASE SNPs are predicted to be deleterious. 95% of these deleterious ASE SNPs are found in only one or two animals. Interestingly, we found one T/C deleterious ASE SNP (chromosome10, position 37,912,737) within ZFP106 in one animal (LIM18). ZFP106 encodes a zinc fingered RNA binding protein. Disruption of *Zfp106* in mice induces several skeletal muscle phenotypic abnormalities^73–75^, such as severe muscle wasting^74^, loss of muscle strength^73–75^ and degeneration of muscle fibers^75^ in homozygous knock out *Zfp106* −/− mice. Heterozygous *Zfp106* +/− mice are comparable to wild type littermates^74,75^. These results suggest that *ZFP106* might not be a dosage-sensitive gene and that haploinsufficiency of *ZFP106* (in ASE SNP heterozygous animals) might not impact muscle physiology. We also found a deleterious ASE SNP (rs110365838) within *MAP4*, a muscle-specific microtubule associated protein which is expressed in early myogenesis^76^ and that is required for muscle cell differentiation^77^. This ASE SNP was detected in two animals (LIM2 and LIM15). We didn’t find, so far, any information on potential consequences of deleterious variants within this gene. However, because of the critical role of *MAP4* in muscle development, it will be interesting to investigate if the two heterozygous animals for this ASE SNP have normal amount of MAP4 protein.

Third, we examined if ASE SNPs could impact genes important for muscle cell development or function. We focused on ASE SNPs located in downstream, upstream, 5′ or 3′ UTR regions, as they might have an effect on the regulation of the transcription of important genes. We found that myogenin (*MYOG*), a muscle-specific transcription factor required to induce myogenesis^78^, had in total 21 ASE SNPs, including 5 and 7 in downstream and 3′UTR regions, respectively. However, disruption of murine myogenin showed no overt effects in heterozygous *Myog* +/− mice^79^ suggesting that a potential reduction of MYOG in animals heterozygous for those 12 ASE SNPs might not have phenotypic consequences.

## Conclusion

We performed a genome-wide survey of ASE using 19 Limousine muscle samples combining WGS and RNA-Seq data. This analysis shows that ASE is pervasive in beef muscle. We identified 5,658 ASE SNPs located in 2,119 genes and 37.2% of these ASE SNPs are found within QTLs associated to meat or carcass traits. We validated 5 out of 6 selected ASE SNPs suggesting that our pipeline identify mostly true ASE SNPs. In addition, we detected SNPs with genotypes significantly associated with gene expression levels.

For example, we identified one SNP in the 3′UTR region of *PRNP* that could be a causal mutation by modifying binding sites of several miRNAs. We showed that our *in silico* ASE approach can facilitate the identification of candidate *cis*-regulatory SNPs. However, further work is needed to validate these candidates. In the future, functional analyses of the impact of polymorphisms within TF or miRNA binding sites will try to elucidate the molecular mechanisms underlying gene expression imbalance.

## Supplementary information

Supplementary informations

Table S1

Table S2

Table S3

Table S4

Table S5

Table S6

Table S7

Table S8

Table S9

Table S10

## Data Availability

RNA-Seq data analysed during the current study is available from the European Nucleotide Archive (accession numbers ERP002220, E-MTAB-2646, E-MTAB-4625 and E-MTAB-6947). The ASE SNPs identified in this study are included in the Table S4.
