# Supplementary material for: Survey of allele specific expression in bovine muscle
Source: Sci Rep. 2019 Mar 12;9:4297. doi: 10.1038/s41598-019-40781-6 (PMC6414783; doi:10.1038/s41598-019-40781-6)
Supplement: Supplementary file 1 — Supplementary informations [file 41598_2019_40781_MOESM1_ESM.pdf]

Survey of allele specific expression in bovine muscle

Gabriel M. Guillocheau, Abdelmajid El Hou, Cédric Meersseman, Diane Esquerré, Emmanuelle Rebours, Rabia Letaief, Morgane Simao, Nicolas Hypolite, Emmanuelle Bourneuf, Nicolas Bruneau, Anne Vaiman, Christy J. Vander Jagt, Amanda J. Chamberlain and Dominique Rocha.

Supplementary data:

Table S1. Primers used for the Pyrosequencing validation.

Table S2. Results of WGS read mapping.

Table S3. Results of RNA-Seq read mapping.

Table S4. Information on the identified ASE-SNPs.

For each animal: # REF, number of reads with the reference allele; # ALT, number of reads with the alternative allele, # Total, total number of reads.

Table S5. Distribution of ASE-SNPs per individual.

Het, heterozygous.

Table S6. Information on ASE-SNPs located within QTL regions.

Table S7. Results of the Gene Ontology terms enrichment analysis.

N, total number of genes; B, total number of genes associated with a particular GO term; n, number of genes in the target set; b, number of genes from the target set associated with the given GO term.

Table S8. Results of the correlation analysis.

Table S9. Information on SNPs impacting miRNA binding sites.

Table S10. Information on muscle expression of miRNA impacted by SNPs.

Expression in muscle tissue is highlighted in green.

Figure S1. Boxplots of significantly correlated SNPs in 3'UTR regions. (N) number of animals per genotype.

Figure S2. Boxplots of significantly correlated SNPs within *AOX1*. (N) number of animals per genotype.

Figure S3. Multispecies alignment of AOX1 protein sequences around the bovine p.G1023C substitution using CLUSTALW.

Species names and sequence accession numbers in NCBI are: cattle (*Bos taurus*, NP_788841.1), bison (*Bison bison bison*, XP_010859241.1), yak (*Bos mutus*, ELR56892.1), sheep (*Ovis aries*, XP_004004859.1), goat (*Capra hircus*, XP_005676396.1), sperm whale (*Physeter catodon*, XP_023980530.1), killer whale (*Orcinus orca*, XP_004262923.1), bottlenose dolphin (*Tursiops truncatus*, XP_019794738.1), Beluga whale (*Delphinapterus leucas*, XP_022423076.1), dromedary (*Camelus dromedarius*, XP_010990253.1), pig (*Sus scrofa*, NP_001295402.1), Sumatran orangutan (*Pongo abelii*, PNJ73708.1), human (*Homo sapiens*, NP_001150.3), chimpanzee (*Pan troglodytes*, NP_001295372.1), California sea lion (*Zalophus californianus*, XP_027445411.1), guinea pig (*Cavia porcellus*, AFG18181.1), mouse

(*Mus musculus*, NP_033806.2), rat (*Rattus norvegicus*, NP_062236.2), chicken (*Gallus gallus*, NP_001033781.1), golden hamster (*Mesocricetus auratus*, XP_021089146.1), turkey (*Meleagris gallopavo*, XP_019472346.1), xenopus (*Xenopus tropicalis*, XP_017953036.1), Japanese quail (*Coturnix japonica*, XP_015723660.1), fugu (*Takifugu rubripes*, XP_003966732.1).

Position of the p.G1023C substitution is indicated by **(*)**. Amino acids different from the bovine sequence are highlight in green.

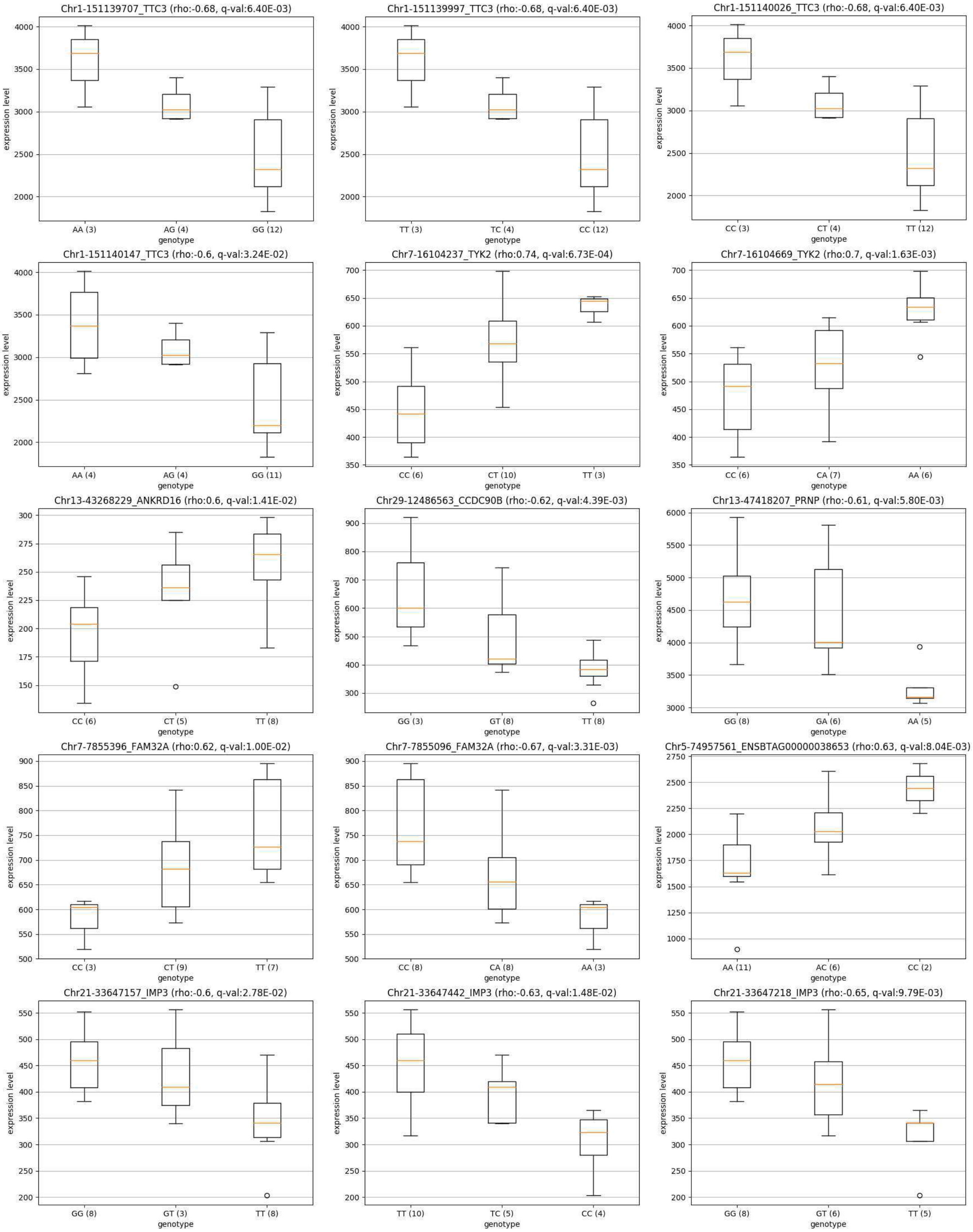

Figure S1. Boxplots of significantly correlated SNPs in 3'UTR regions. (N) number of animals per genotype.

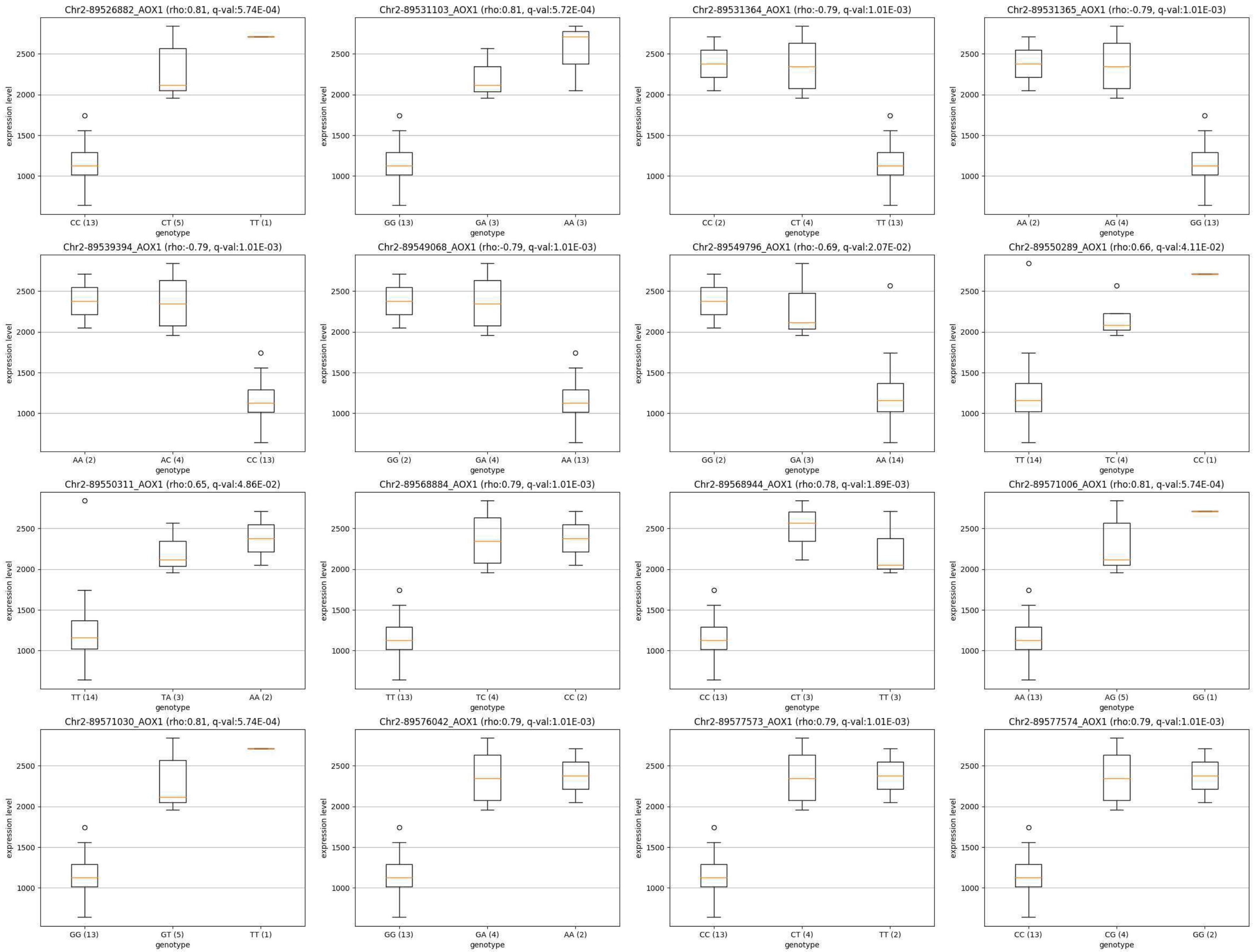

Figure S2. Boxplots of significantly correlated SNPs within *AOX1*. (N) number of animals per genotype.

Figure S3

```
                                        p.G1023C

                                          (*)
fugu                 KRGMSIIPIKYGIGFSESSLNQAAALVHIYKDGSVLVTHGG
xenopus              KKGIAIIPLKFTVGFVEKTYHQAAALVHIYRDGYVLVSHSG
Japanese_quail       KKGIAIVPMKFPFGLGSRYLSQAAALVHIYTDGSVLLTHGG
chicken              KKGIAIVPMKFPFGLGSRYLSQAAALVHIYTDGSVLLTHGG
turkey               KKGIAIVPMKFPFGLGSRYLSQAAALVHIYTDGSVLLTHGG
guinea_pig           KRGLAMVPLKFPVGLGSVAMGQAAALVHVYLDGSVLLTHGG
golden_hamster       KRGLAMIPLKFPVGIGSVAMGQAAALVHIYLDGSALVTHGG
mouse                KRGMAVIPLKFPVGIGSVAMGQAAALVHIYLDGSALVSHGG
rat                  KRGMAVIPLKFPVGVGSVAMGQAAALVHIYLDGSALVSHGG
Sumatran_orangutan   KKGLAMVPLKYPVGLGSRAAGQAAALVHIYLDGSVLVTHGG
human                KKGLAMVPLKFPVGLGSRAAGQAAALVHIYLDGSVLVTHGG
chimpanzee           KKGLAMVPLKFPVGLGSRAAGQAAALVHIYLDGSVLVTHGG
California_sea_lion  KKGLAMVPLKFPVGLGSVAMGQAAALVHIYLDGSVLVTHGG
goat                 KKGLAMVPLKFPVGVGSVAAGQAAALVHIYLDGSVLVTHGG
sheep                KKGLAMVPLKFPVGVGSVAAGQAAALVHIYLDGSVLVTHGG
yak                  KKGLAMVPLKYPIGLGSVAAGQAAALVHIYLDGSVLVTHGG
cattle               KKGLAMVPLKYPIGLGSVAAGQAAALVHIYLDGSVLVTHGG
bison                KKGLAMVPLKYPIGLGSVAAGQAAALVHIYLDGSVLVTHGG
pig                  KKGLAMVPLKYPVGTGSLAMGQAAALVHIYLDGSVLVTHGG
dromedary            KKGLAMVPLKFPVGLGSTAAGQAAALVHIYLDGSVLVTHCG
sperm_whale          KKGLAMVPLKFPVGLGSVAAGQAAALVHIYLDGSVLVTHGG
killer_whale         KKGLAMVPLKFPVGLGSVAAGQAAALVHIYLDGSVLVTHGG
bottlenose_dolphin   KKGLAMVPLKFPVGLGSVAAGQAAALVHIYLDGSVLVTHGG
Beluga_whale         KKGLAVVPLKFPVGLGSVAAGQAAALVHIYLDGSVLVTHGG
                     * *    * *   *       ******* * **  *  * *
```
